# Pancreas Size and Fat Content Increase during Pregnancy and Correlate with Glycemic Control

**DOI:** 10.1055/a-2831-5762

**Published:** 2026-03-23

**Authors:** Elizabeth M. Widen, Sara Dube, Ingrid Harris, Madeline van Heukelum, Josephine Do, Aidan Dulaney, Isaiah Janumala, Jeffrey J. Luci, Lorie M. Harper, Alison G. Cahill, John Virostko

**Affiliations:** 1 Department of Nutritional Sciences, University of Texas, Austin, Texas, United States; 2 Department of Women's Health, Dell Medical School, University of Texas, Austin, Texas, United States; 3 Department of Pediatrics, Dell Medical School, University of Texas, Austin, Texas, United States; 4 Department of Diagnostic Medicine, Dell Medical School, University of Texas at Austin, Austin, Texas, United States; 5 Center for Advanced Human Brain Imaging Research, Rutgers University, Piscataway, New Jersey, United States; 6 Scully Neuroimaging Center, Princeton University, Princeton, New Jersey, United States; 7 Oden Institute for Computational Engineering and Sciences, The University of Texas at Austin, Austin, Texas, United States; 8 Livestrong Cancer Institutes, Dell Medical School, University of Texas at Austin, Austin, Texas, United States

**Keywords:** imaging, kidney, lactation, liver, MRI, spleen, volume

## Abstract

**Objective:**

The pancreas plays a critical role in adapting to the increased metabolic demands and insulin resistance of pregnancy. In rodents, beta cell proliferation during pregnancy induces pancreas growth, but whether similar changes occur in the human pancreas remains unknown. This study characterizes longitudinal changes in the size and composition of the pancreas during pregnancy and postpartum, and examines correlations with breastfeeding, adiposity, and metabolic assays.

**Study Design:**

Sixty-one pregnant women were followed up to three visits during pregnancy (15, 25, and 35 weeks) and three postpartum (6 weeks, 6 months, and 12 months). MRI was conducted to assess pancreas, spleen, and kidney size as well as pancreas, liver, and whole-body adiposity. Glucose tolerance testing was performed at 24 to 28 weeks' gestation. In a subset of participants ( *n* = 58), additional glucose assays were performed at 15 weeks' gestation. Pancreas size was normalized for concurrently measured body weight to calculate pancreas volume index (PVI).

**Results:**

The size of the pancreas increased by approximately 20% from 15 to 35 weeks' gestation ( *p* < 0.001) and declined postpartum. PVI was positively correlated with whole-body fat mass ( *R* ^2^ = 0.12) and fasting blood glucose ( *R* ^2^ = 0.18) at 15 weeks' gestation. Higher pancreas fat content at 15 weeks predicted impaired response to glucose challenge 10 weeks later ( *R* ^2^ = 0.33). PVI was low, and pancreatic fat was high at 15 weeks in two participants who were subsequently diagnosed with gestational diabetes mellitus. Any breastfeeding 1-year postpartum correlated with a smaller decline in PVI from 35 weeks' gestation to 1-year postpartum ( *p* < 0.05).

**Conclusion:**

Pancreas size and fat content change dynamically during pregnancy and postpartum, and correlate with glucose levels, prenatal adiposity, and breastfeeding.

**Key Points:**

- The pancreas adapts to the metabolic demands of pregnancy by increasing in size and shrinking postpartum.
- Small pancreas size and high pancreas fat content early in pregnancy may predict subsequent abnormal glucose tolerance.
- Breastfeeding 1-year postpartum was associated with a smaller decline in pancreas size compared to those who were not breastfeeding.

## Introduction

The pancreas plays an integral role in maintaining glucose homeostasis by producing hormones, namely insulin secreted from the pancreatic beta cell, that regulate glucose absorption and secretion. Pregnancy is accompanied by altered maternal metabolism, including insulin resistance and changes in hormone levels regulating glucose metabolism. Hyperglycemia during pregnancy can increase the risk of pregnancy complications, including gestational diabetes mellitus (GDM), preeclampsia, preterm delivery, and excessive fetal growth. Rodent studies indicate that pancreas size increases during pregnancy and lactation, declines after weaning, and correlates with beta cell proliferation. 1 However, as beta cell proliferation during pregnancy is thought to be more limited in humans, 2 it is not known whether pancreas growth dynamics are similar in human pregnancy or postpartum.

Radiological imaging of the human pancreas can detect dynamic changes in pancreas size and composition. The size of the pancreas increases during childhood and adolescence and declines in older adults. 3 4 Furthermore, pancreas imaging correlates with altered metabolism. For instance, the pancreas is smaller in individuals with both type 1 (T1D) and type 2 diabetes (T2D) and displays increased fat content in T2D. 5 Alterations to the pancreas likely precede diagnosis with these diseases, as pancreas size declines prior to diagnosis with T1D 6 and pancreatic fat content is increased in individuals predisposed to T2D. 7 The mechanisms underlying these changes in the pancreas are unclear, but it is thought that pancreas size is influenced by insulin action 8 and pancreatic beta cell mass. 9 Interestingly, longitudinal studies have shown that pancreas size is dynamic, decreasing after diagnosis with diabetes 10 but increasing in response to diabetes remission. 11 Taken together, these findings suggest that imaging of pancreas size may provide insight into pancreatic beta cell mass and/or function.

This study sought to longitudinally measure pancreas size and fat content over the course of pregnancy and postpartum. We hypothesized that pancreas size would increase during pregnancy in response to beta cell hypertrophy and proliferation and return to pre-pregnancy size post-weaning. Concurrent changes in the volume of the kidney and spleen were assessed to determine whether changes observed in the pancreas also occurred in other abdominal organs. We further examined the relationship between maternal pancreas dynamics with glucose metabolism, adiposity, and breastfeeding status postpartum.

## Materials and Methods

### Study Participants

This is an ancillary study of an observational cohort study, the Mother Infant NuTrition Study (MINT; *n* = 61; PI: E.M.W.), that was designed to assess adipose tissue changes and their determinants across pregnancy and postpartum. All participants in the MINT study were included in this ancillary study of the pancreas. The study was originally designed as a pilot study with a sample size of 40, but the size was increased due to the COVID-19 pandemic's impact on data collection. Further details on this cohort have been previously published. 12 Briefly, pregnant individuals aged 18 and older with singleton pregnancies <14 weeks' gestation were recruited through local obstetric providers, social media, and snowball sampling. Exclusion criteria included BMI ≥35 kg/m ^2^ (necessary to fit within the MRI field of view in late pregnancy), preexisting diabetes, known congenital anomaly, recent weight loss of >5% body weight prior to pregnancy, prior weight loss surgery, or implants contraindicated for MRI. Participants completed up to six study visits. Three study visits occurred during pregnancy at approximately 15 (range 13–17) weeks' gestation, approximately 25 (range 23–27) weeks' gestation, and approximately 35 (range 33–37) weeks' gestation. These visits were designed to assess changes in adiposity from the early to late second trimester and then close to delivery. Three study visits occurred postpartum at 6 weeks, 6 months, and 12 months. The approximate timing of each MRI is shown in Table 1 . Participants' height was measured at enrollment with a stadiometer, and weight was measured with a digital scale, Seca 874, at each visit. At postpartum visits, breastfeeding status was recorded at each visit via questionnaire. This study was registered through ClinicalTrials.gov (NCT04132310) and approved by the UT Austin Institutional Review Board. This study adheres to the STROBE statement guidelines for reporting observational studies.

**Table 1:** Mother Infant Nutrition cohort study participant characteristics in the pancreas substudy

Characteristics	*N* = 61
Age	33.0 years (± 4.2); max: 45, min: 24
Hispanic	10 (16%)
Race	White: 49 (80%) Asian: 5 (8%) Multiracial: 5 (8%) Black: 1 (2%) Unknown: 1 (2%)
Parity	0: 38 (62%) 1: 17 (28%) 2: 6 (10%)
Pre-pregnancy BMI	25.0 (± 4.0); max: 36.5, min: 18.6 Normal: 36 (59%) Overweight: 17 (28%) Obesity: 8 (13%)
Gestational age at MRI 1 (wk)	15.0 (± 0.8); max: 17.9, min: 13.3
Gestational age at MRI 2 (wk)	24.9 (± 0.8); max: 27.0, min: 23.3
Gestational age at MRI 3 (wk)	35.0 (± 0.8); max: 37.0, min: 33.6
Weeks postpartum at MRI 4	6.7 (± 0.6); max: 8.1, min: 5.3
Weeks postpartum at MRI 5	29.3 (± 2.0); max: 33.9, min: 26.5
Weeks postpartum at MRI 6	54.6 (± 2.6); max: 64.4, min: 50.0
Fasting blood glucose at MRI 1	83.7 (± 7.9); max: 104, min: 53
HbA1c at MRI 1	4.58 (± 0.39); max: 5.9, min: 4.0
Breastfeeding at 12 months postpartum ( *N* = 40)	19 (48%)

Abbreviations: BMI, body mass index; HbA1c, glycated hemoglobin; MRI, magnetic resonance imaging.

All values are expressed as mean (± SD) or *N* (percentage).

### Glucose Metabolism Assays

All study participants received either a 1- or 3-hour oral glucose tolerance test between 24 and 28 weeks' gestation as part of standard-of-care monitoring for GDM at their obstetric provider. A majority of participants (93%) completed a fasting blood draw at the initial study visit at 15 weeks' gestation. Glycated hemoglobin (HbA1c) was measured using an A1CNow+ System, and fasting glucose was measured using a HemoCue Glucose 201 system. C-peptide and insulin were assessed in a subsample at the Atherosclerosis Clinical Research Lab at Baylor College of Medicine. C-peptide and insulin were measured via electrochemiluminescence immunoassay using a Roche Cobas e411 in EDTA plasma.

### Magnetic Resonance Imaging

MRI was performed on a 3-T Siemens Vida scanner using a standardized protocol validated for quantitative pancreas evaluation across imaging centers and hardware. 13 Abdominal imaging was performed in the axial plane at 1.5 × 1.5 × 4 mm spatial resolution with a field of view spanning the dome of the liver extending below the head of the pancreas. Images were acquired during breath-holds to minimize respiratory motion. Acquisitions included a fat-suppressed T1-weighted ultrafast gradient echo sequence and a T2-weighted fast-spin echo sequence. A three-dimensional 6-echo Dixon acquisition was collected to calculate fat fraction. A whole-body multistation T1-weighted MRI was also acquired to calculate whole-body composition. 14 The whole-body MRI protocol includes acquisition of approximately 40 axial slices (10 mm thickness at 40 mm intervals) spanning the entire body with participant arms extended above the head and breath-holds for abdominal sections.

### Image Analysis

The pancreas was outlined on each T1-weighted slice by a reader blinded to any clinical or metabolic information. On MRI scans that also included the entire spleen, left kidney, or right kidney in the field of view, the respective organ was outlined by a blinded reader. Regions of interest delineating each organ were created using HOROS software (Annapolis, MD) to calculate organ volume. Pancreas volume index (PVI) was calculated by dividing the pancreas volume by the participant's concurrently measured body weight to account for interindividual differences in body habitus, as previously described. 15 For fat fraction measurements, a map of the abdominal fat fraction was calculated using Siemens LiverLab software (Siemens Healthineers, Erlangen, Germany). Circular regions of interest were placed on the fat fraction map over the liver and pancreas head, body, and tail to measure fat fraction in each region, taking care to avoid the pancreatic duct or image artifacts. SliceOmatic image analysis software (Tomovision, Montreal, CA) was used to determine body composition components from the whole body acquisition, including total adipose tissue, which is estimated from MRI volumes and converted to mass using an assumed density of 0.92 g/cm ^3^ for adipose tissue. 16 Body fat percentage was calculated as fat mass/body mass.

### Statistical Analysis

Statistical analysis was performed using R (version 4.1.1). Pairwise comparisons of independent groups were assessed using Student's *t* -test. Associations between independent variables were evaluated using Pearson correlation. Longitudinal data were analyzed using a linear mixed-effects model with each participant set as a random factor, that is, subject-specific random intercept. Continuous data are presented as mean ± standard deviation. An alpha of 0.05 was used for all statistical analyses. Because these analyses were exploratory and hypothesis-generating, we did not apply formal corrections for multiple comparisons.

## Results

A total of 61 pregnant women were enrolled in this study. Participant demographics are shown in Table 1 . All 61 participants completed MRI at 15 weeks' gestation, 42 participants completed MRI at 25 weeks' gestation, 36 participants completed MRI at 35 weeks' gestation, 31 participants completed MRI at 6 weeks' postpartum, 12 participants completed MRI at 6 months' postpartum, and 40 participants completed MRI at 12 months' postpartum.

**Fig. 1:**
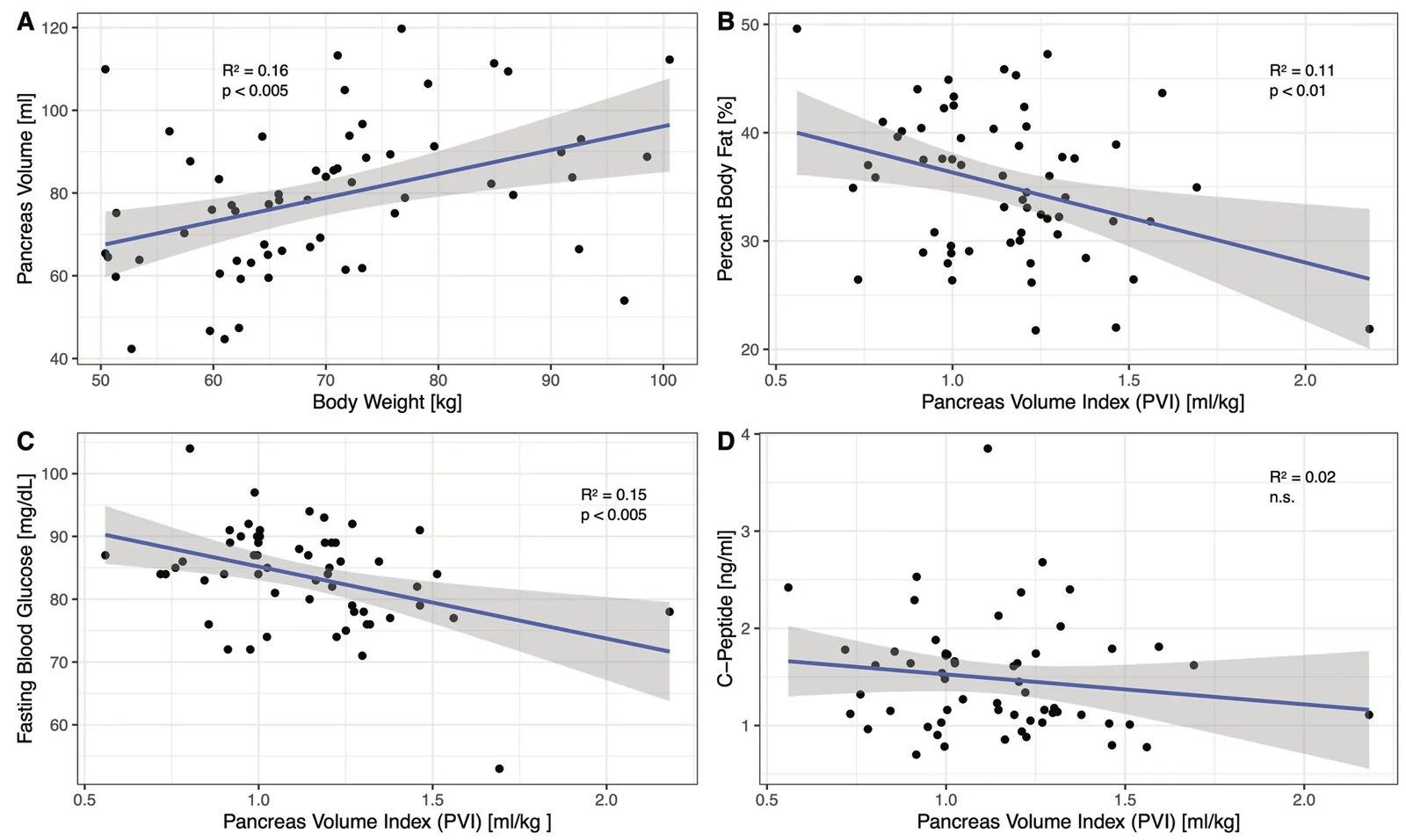
( **A** ) The volume of the pancreas correlates with body weight ( *N* = 61). ( **B** ) When normalized for body weight, the pancreas volume index (PVI) inversely correlates with body fat percentage ( *N* = 61). ( **C** ) PVI inversely correlates with fasting blood glucose ( *N* = 57). ( **D** ) PVI does not correlate with C-peptide levels ( *N* = 33). All measurements were performed at approximately the 15th week of pregnancy.

**Fig. 2:**
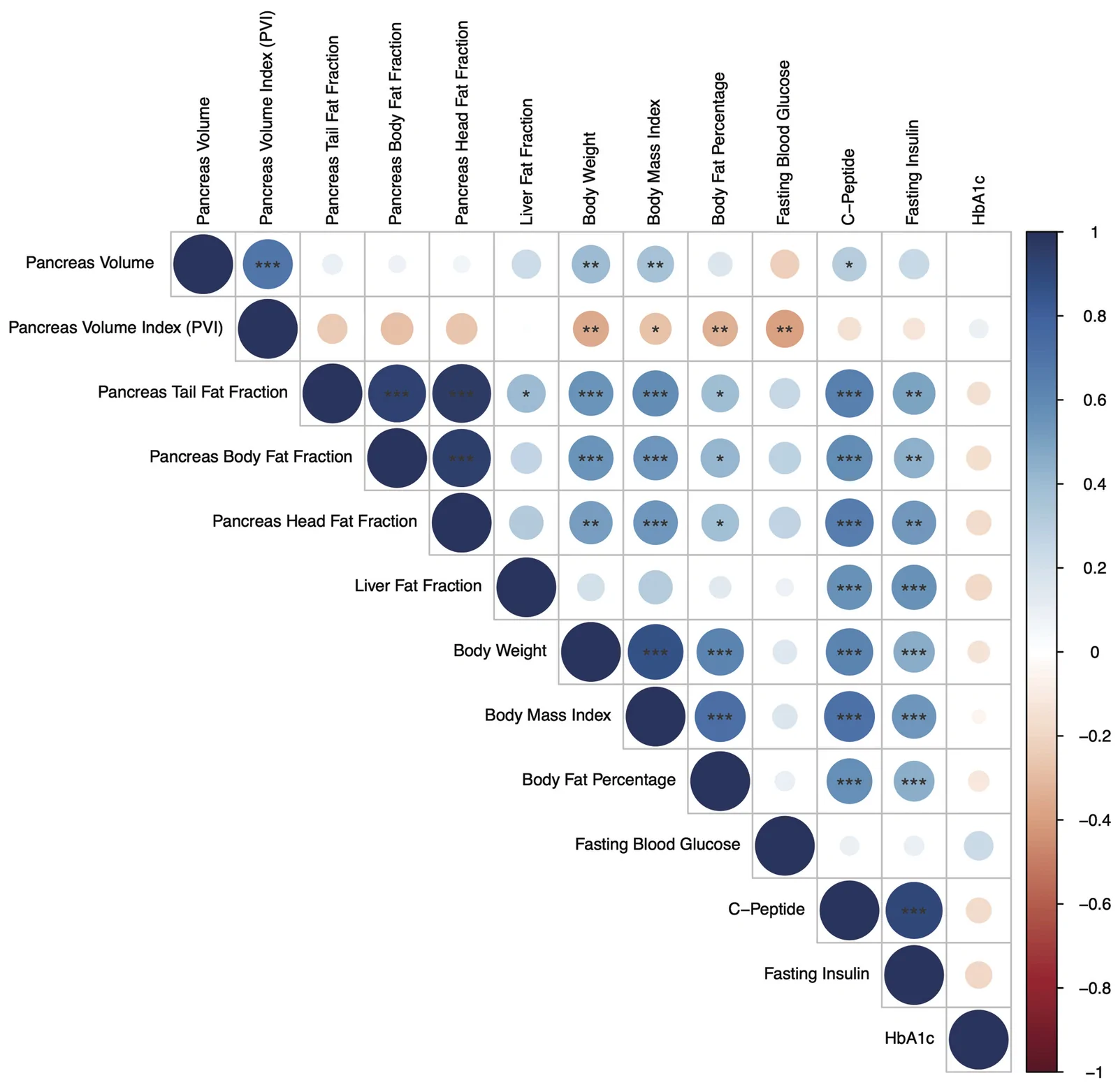
Correlation plot of pancreas size, pancreas and liver fat fraction, and metabolic blood testing performed at the 15th week of pregnancy. The color of each circle represents the direction of correlation (blue is positive, red is negative) while the size of each circle corresponds with the magnitude of correlation (larger circles are stronger correlations). Statistically significant correlations are noted with asterisks: ***, *p* < 0.001; **, *p* <0 .01; *, *p* < 0.05. HbA1c, glycated emoglobin.

At the first MRI at 15 weeks' gestation, pancreas volume correlated with body weight ( *R* ^2^ = 0.16, *p* < 0.005, Fig. 1A ). To account for interindividual differences in habitus, we normalized pancreas volume by body weight to yield a PVI, as previously described. 17 PVI at 15 weeks' gestation was inversely correlated ( *R* ^2^ = 0.11, *p* < 0.01) with body fat percentage (i.e., women with higher adiposity had a lower pancreas volume normalized for body weight) as shown in Fig. 1B . PVI was also negatively correlated with fasting blood glucose ( *R* ^2^ = 0.15, *p* < 0.005, Fig. 1C ). The association between PVI and fasting blood glucose remained significant after adjustment for BMI ( *R* ^2^ = 0.23, *p* < 0.01). PVI did not correlate with C-peptide ( *p* = 0.28, Fig. 1D ) with or without adjustment for BMI. PVI and pancreas volume were also not correlated with concurrently assessed fasting insulin nor HbA1c ( Fig. 2 ).

Fat content measurements in different organs correlated with one another at 15 weeks' gestation. Pancreatic fat content in the head, body, and tail of the pancreas was highly correlated. Pancreas and liver fat content were moderately correlated in the pancreas tail ( *p* < 0.05, Fig. 2 ), but liver fat did not correlate with fat content of the pancreas head or body. Pancreas fat in all regions was positively correlated with body weight ( *p* < 0.001), BMI ( *p* < 0.001), and body fat percentage ( *p* < 0.05, Fig. 2 ). Fat fractions in the liver and all pancreas regions were positively associated with C-peptide and fasting insulin ( Fig. 2 , *p* < 0.001 and *p* < 0.01, respectively). We found no correlations between pancreas or liver fat with fasting blood glucose or HbA1c ( Fig. 2 ).

**Fig. 3:**
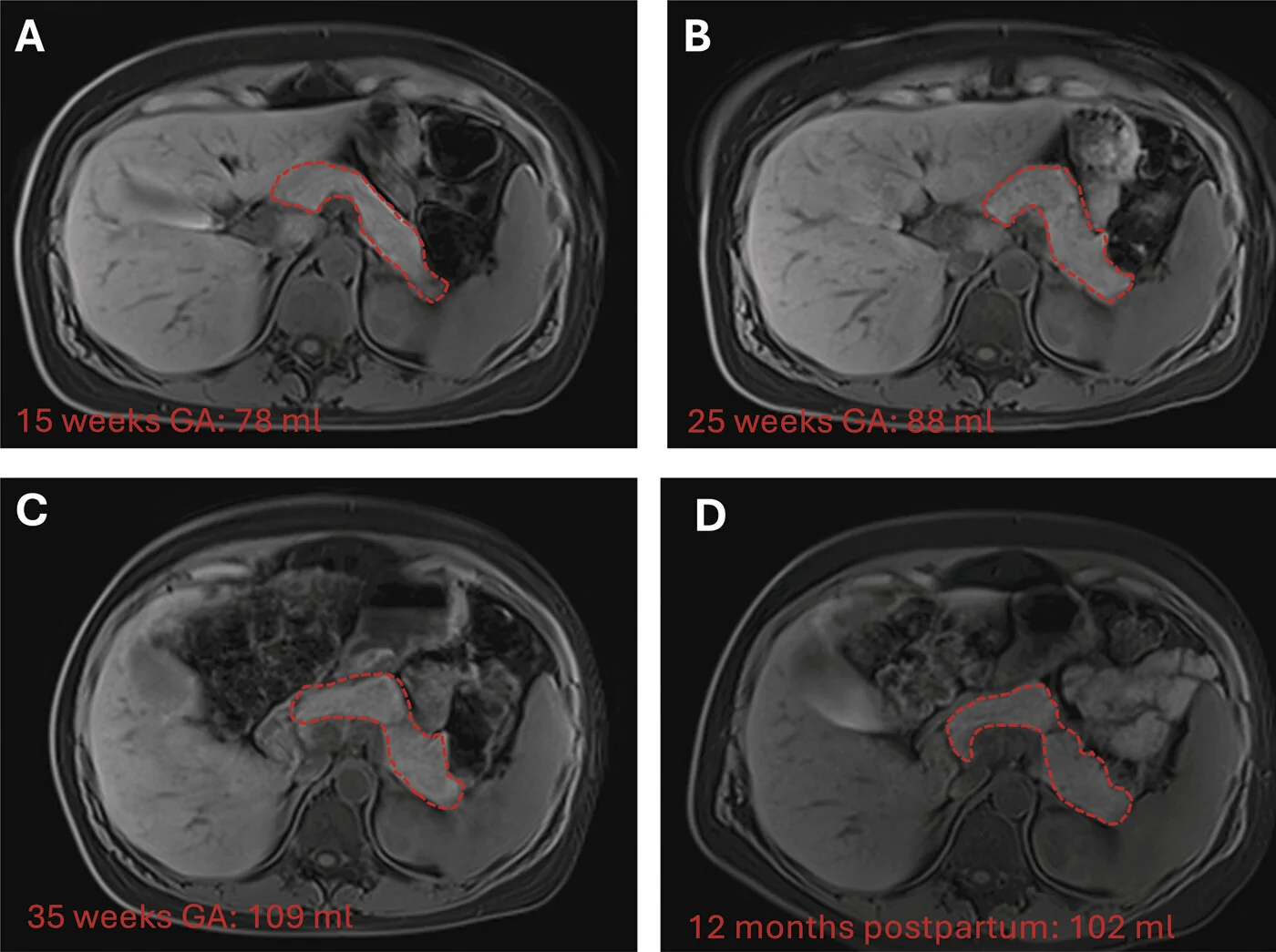
Longitudinal MRI scans of a representative study participant acquired at ( **A** ) 15 weeks' gestation, ( **B** ) 25 weeks' gestation, ( **C** ) 35 weeks' gestation, and ( **D** ) 12 months' postpartum. The pancreas is outlined in red on each axial MRI slice and the pancreas volume is noted on each image. The pancreas volume increased over the course of pregnancy from 78 mL to 109 mL and declined postpartum to 102 mL.

**Fig. 4:**
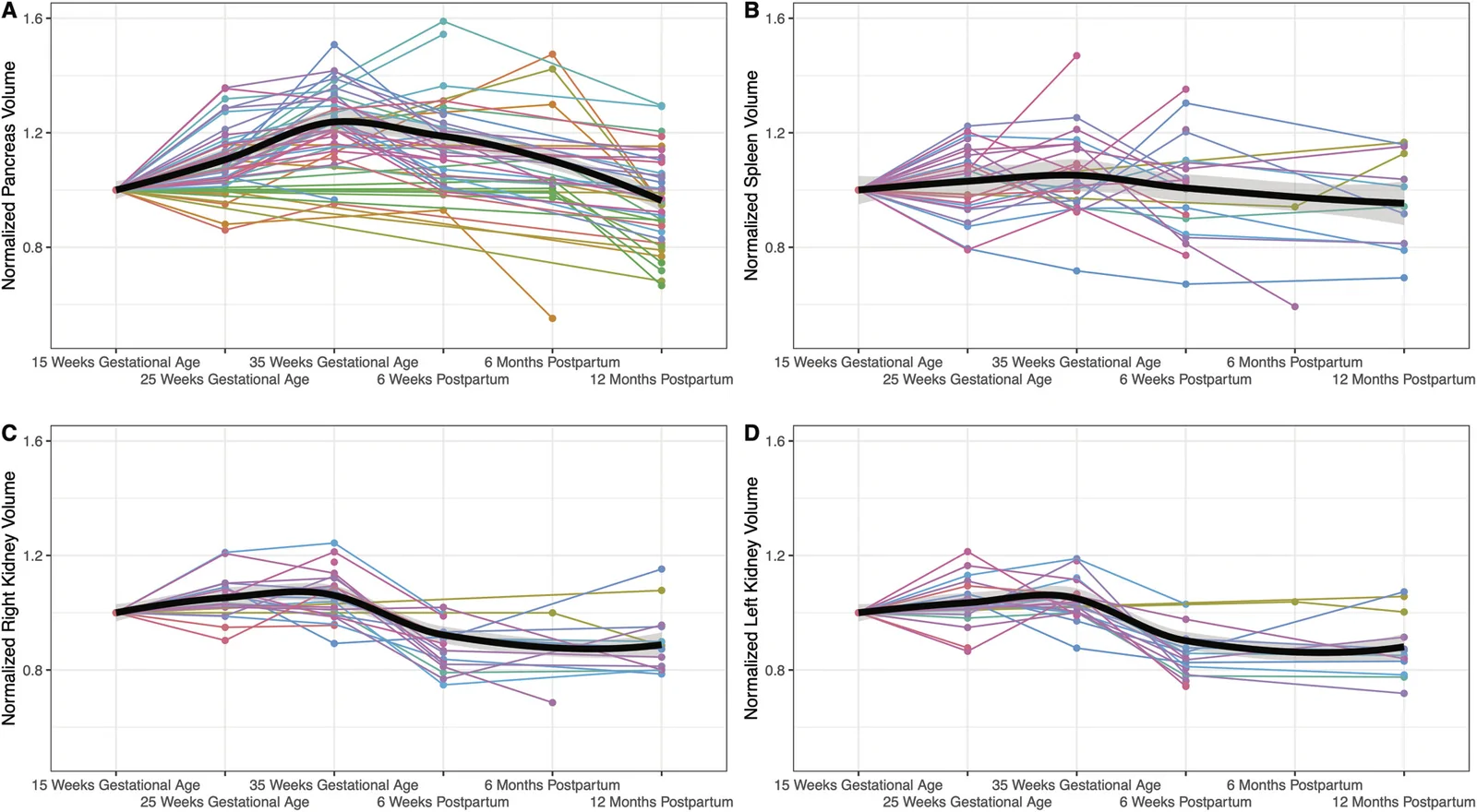
( **A** ) The volume of the pancreas increases over the second and third trimester of pregnancy and declines postpartum ( *N* = 61). ( **B** ) The volume of the spleen does not increase over the second and third trimester of pregnancy or decline postpartum ( *N* = 29). The volume of the ( **C** ) right ( *N* = 27) and ( **D** ) left kidney ( *N* = 27) increase over the second and third trimester of pregnancy and declines postpartum. For each abdominal organ, each participant's longitudinal trajectory is color-coded, and pancreas volume is normalized to its value at 15 weeks' gestational age. The group average and 95% CI are shown with a black line and gray shading, respectively.

Longitudinal imaging of the pancreas revealed dynamic changes over the course of pregnancy and postpartum. Representative longitudinal abdominal MRI images of a study participant are shown in Fig. 3 . Pancreas volume increased by 20% on average from 15 weeks' gestation to 35 weeks' gestation ( *p* < 0.001, Fig. 4A ), followed by a commensurate decline in pancreas volume postpartum. We also measured spleen volume in 57% of MRI scans, right kidney volume in 54% of scans, and left kidney volume in 52% of scans (the percentage of scans when these organs were in the field of view). No changes in the volume of the spleen during pregnancy or postpartum were detected ( Fig. 4B ). The volume of the right ( *p* < 0.001, Fig. 4C ) and left kidney ( *p* < 0.01, Fig. 4D ) increased during pregnancy followed by a postpartum decline to a volume lower than the measurement at 15 weeks' gestation.

**Fig. 5:**
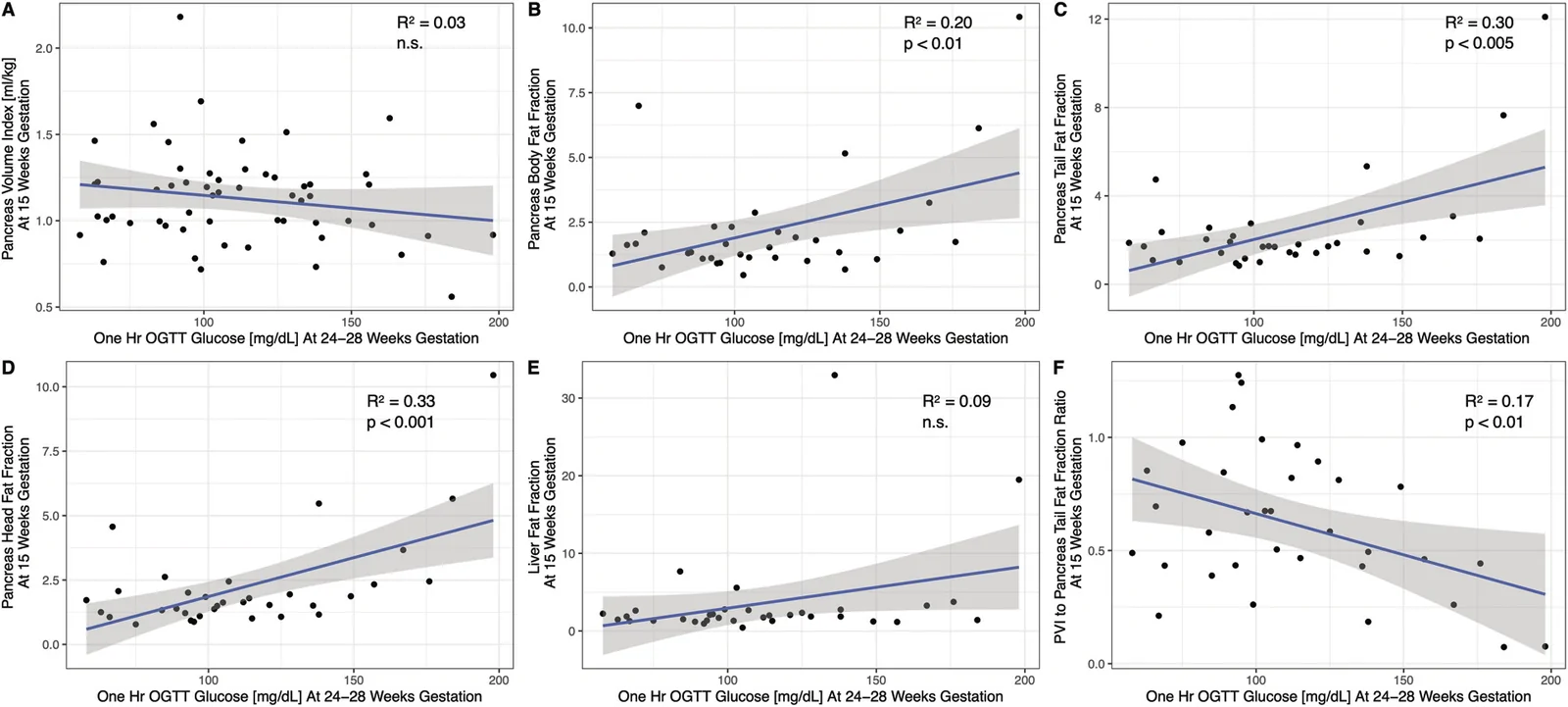
( **A** ) Pancreas volume index at 15 weeks' gestation does not correlate with glucose tolerance testing at 24 to 28 weeks' gestation ( *N* = 56). Fat content of the ( **B** ) pancreas body, ( **C** ) tail, and ( **D** ) head at 15 weeks' gestation all correlate with glucose tolerance testing at 24 to 28 weeks' gestation ( *N* = 35). ( **E** ) Liver fat content at 15 weeks' gestation does not correlate with glucose tolerance testing at 24 to 28 weeks' gestation ( *N* = 35). ( **F** ) A ratio of PVI divided by the pancreas tail fat fraction at 15 weeks' gestation correlates with glucose tolerance testing at 24 to 28 weeks' gestation ( *N* = 35). PVI, pancreas volume index.

We explored whether MRI measurements of pancreas size and pancreas and liver fat content at 15 weeks' gestation could predict glucose tolerance testing performed between 24 and 28 weeks' gestation. PVI ( Fig. 5A ) measured at 15 weeks' gestation trended smaller, but did not reach statistical significance, in participants with impaired glucose tolerance testing performed approximately 10 weeks later, including when adjusting for BMI. Fat content of the pancreas body ( Fig. 5B ), tail ( Fig. 5C ), and head ( Fig. 5D )—all inversely correlated with glucose tolerance testing performed approximately 10 weeks later ( *p* < 0.01, all). After adjusting for BMI, the fat content of the tail and head remained inversely correlated with glucose tolerance ( *p* < 0.05), but the pancreatic body fat content did not ( *p* = 0.09). Liver fat content ( Fig. 5E ) did not correlate with glucose tolerance testing performed approximately 10 weeks later ( *p* = 0.07), including when adjusting for BMI ( *p* = 0.22). Reasoning that PVI tended to inversely correlate with glucose levels while pancreas tail fat fraction directly correlated with glucose levels, we divided PVI by the pancreas tail fat fraction. This PVI to pancreas tail fat fraction ratio inversely correlated with glucose tolerance testing performed approximately 10 weeks later ( Fig. 5F ), although this correlation was attenuated after adjustment for BMI ( *p* = 0.15).

**Fig. 6:**
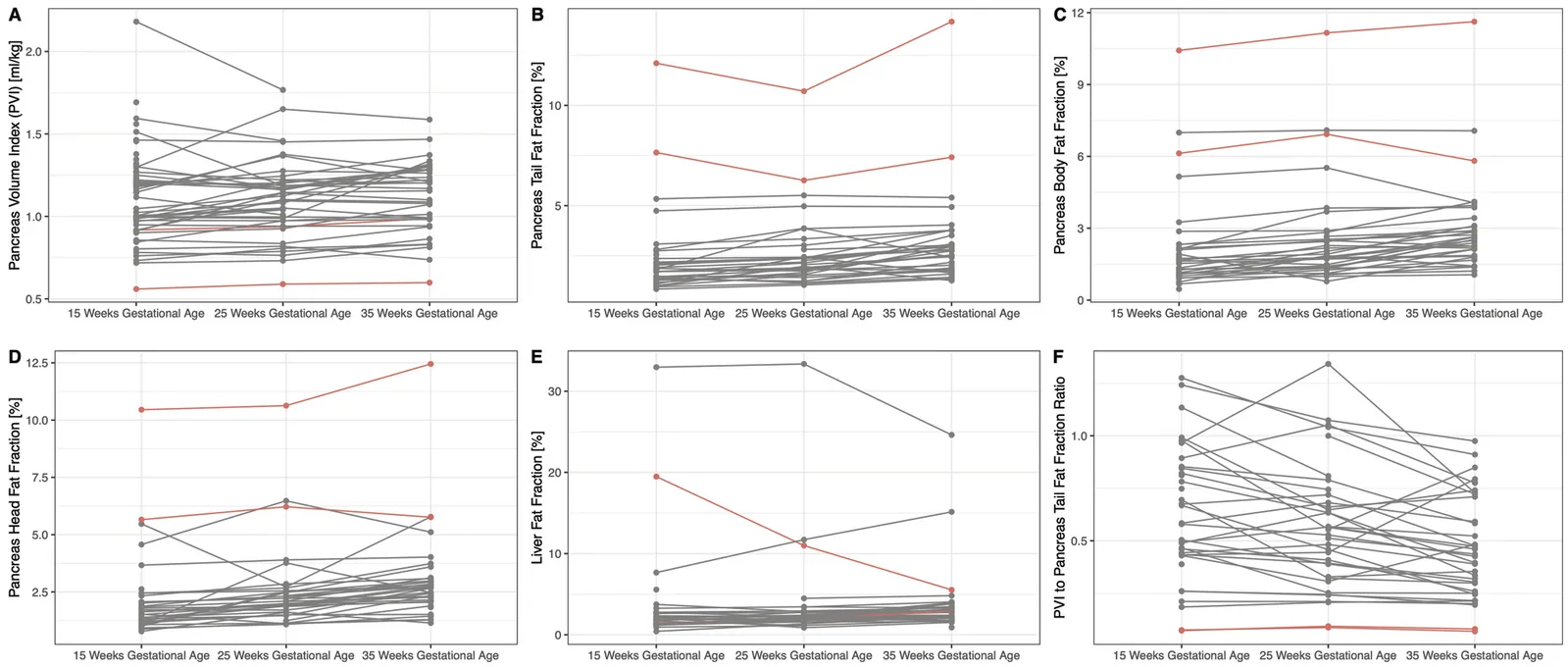
( **A** ) Pancreas volume index (PVI) increases over the course of pregnancy in mothers who do not develop GDM ( *N* = 56) as well as two who developed GDM ( *N* = 2, shown in red). Pancreas fat fraction in the ( **B** ) pancreatic tail, ( **C** ) body, and ( **D** ) head increases over the course of pregnancy in mothers who do not develop GDM ( *N* = 35) and is elevated in mothers who develop GDM ( *N* = 2). ( **E** ) In contrast, liver fat fraction does not change over the course of pregnancy ( *N* = 35) and exhibits no relationship with GDM status. ( **F** ) A ratio of the PVI to the pancreas tail fat fraction is lowest in the two mothers who developed GDM at all MRI scans performed during pregnancy (N = 35). GDM, gestational diabetes mellitus.

Over the course of the study, two participants developed GDM. Both cases of GDM were diagnosed by standard-of-care screening following the second-study MRI. Neither of the mothers who developed GDM had elevated fasting blood glucose (87 and 89 mg/dL, respectively) nor HbA1c (4.5 and 4.4, respectively) at 15 weeks' gestation. Participants exhibited an increase in PVI over pregnancy regardless of GDM status ( Fig. 6A ). One of the participants who developed GDM had the lowest PVI at 15 weeks out of all MRI scans performed in this study, while the other was in the lowest quintile of PVI measurements at 15 weeks. Fat fraction measurements of the pancreas tail were highest in the two mothers who developed GDM across all MRI scans ( Fig. 6B ). Fat fraction measurements of the pancreas body ( Fig. 6C ) and head ( Fig. 6D ) were always highest for one of the participants who developed GDM and were second or third highest for the other participant who developed GDM. While pancreas fat measurements in all three pancreas regions increased for mothers who did not develop GDM ( *p* < 0.001), no changes in pancreas fat fraction were detected in the two mothers who developed GDM. Liver fat fraction increased over the course of pregnancy ( Fig. 6E , *p* < 0.001) but was not notably different in the two mothers who developed GDM. A ratio of PVI divided by the pancreas tail fat fraction was lowest at all study visits for the two mothers who developed GDM ( Fig. 6F ).

**Fig. 7:**
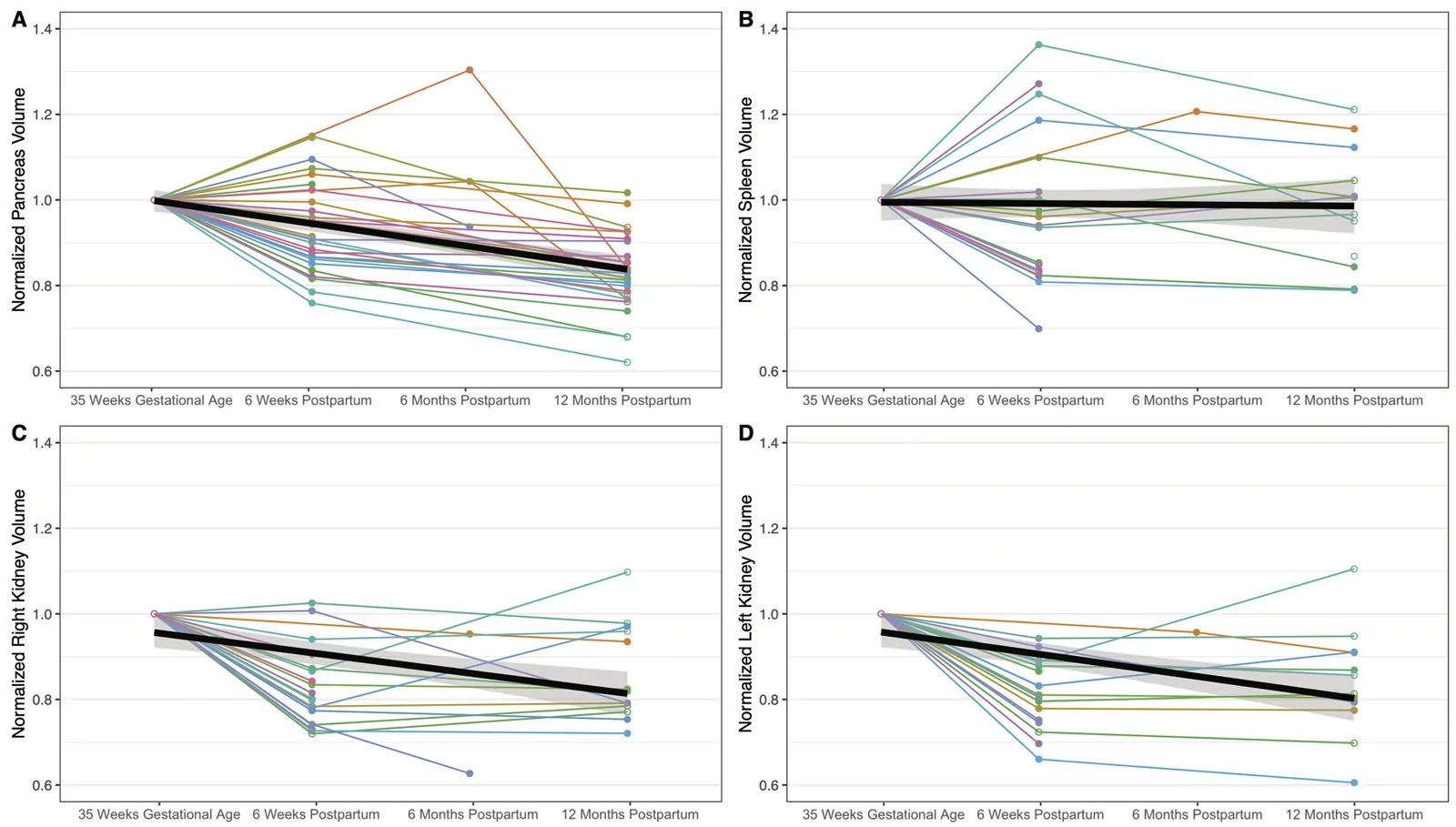
( **A** ) The volume of the pancreas declines postpartum. Each participant's longitudinal trajectory is color-coded, and pancreas volume is normalized to its value at 35 weeks' gestational age ( *N* = 36). Women who were breastfeeding 1-year postpartum (signified by a closed circle) had higher normalized pancreas volume than those who were no longer breastfeeding (open circles). ( **B** ) The volume of the spleen does not change postpartum and is not influenced by breastfeeding status ( *N* = 26). The volume of the ( **C** ) right ( *N* = 25) and ( **D** ) left kidney ( *N* = 24) both decline postpartum, but neither is influenced by breastfeeding status. In each panel, the group average and 95% CI are shown with a black line and gray shading, respectively. Breastfeeding status at each MRI session is shown with a closed circle to indicate current breastfeeding and an open circle indicating no longer breastfeeding at the time of the MRI.

Pancreas volume declined postpartum. Across the entire cohort, pancreas volume decreased by approximately 20% from 35 weeks' gestation to 1-year postpartum ( *p* < 0.001, Fig. 7A ). Mothers who were no longer breastfeeding 1-year postpartum displayed a greater reduction in pancreas volume than those who were still breastfeeding (25% vs. 13%, *p* < 0.05). Spleen volume did not notably decline postpartum and was not associated with breastfeeding status 1-year postpartum ( Fig. 7B ). The volume of the right ( Fig. 7C ) and left ( Fig. 7D ) kidney both declined postpartum ( *p* < 0.001) but were unaffected by lactation status 1-year postpartum.

## Discussion

This study investigated how the size and composition of the pancreas, an organ closely involved in metabolic alterations accompanying pregnancy, change over the course of pregnancy and postpartum using MRI. We found that the pancreas increased in size by approximately 20% from the early second through the third trimester and returned to its initial size 1-year postpartum. Pancreas size and fat content showed correlations with measures of glucose metabolism, with a smaller, fattier pancreas associated with dysglycemia. Notably, participants with a high pancreas fat content later displayed impaired glucose tolerance, two of whom developed GDM, suggesting that pancreas imaging may predict metabolic dysfunction.

Pregnancy induces compensatory beta cell hypertrophy and proliferation in response to altered metabolic demands, hormonal milieu, and insulin resistance, 18 followed by a significant reduction in beta cell mass postpartum. 19 Pregnancy-associated pancreas growth may be related to beta cell proliferation, with a return to pre-pregnancy beta cell mass (and decreased pancreas size) postpartum. 19 20 The magnitude of the increase in pancreas size (20%) is less than the increase in beta cell mass found in autopsy studies (40%). 2 We note that our first MRI occurred at 15 weeks' gestation, at which point some pancreas growth may have already occurred. However, rodent studies indicate that most increases in beta cell mass occur late in pregnancy, 21 although rodent pregnancy differs significantly from human pregnancy in gestation length and fetal maturity. Furthermore, the pancreas size measured 12 months postpartum was similar to that seen at 15 weeks' gestational age, further suggesting there is minimal pancreas growth in early pregnancy. In contrast, kidney size at 15 weeks' gestational age was higher than 12 months postpartum, suggesting kidney growth in the first trimester. Notably, the increase in pancreas size found in our study surpassed any changes in spleen or kidney volume, suggesting that the changes observed in the pancreas exceed those in other abdominal organs.

Longitudinal monitoring of the maternal pancreas may provide novel metabolic information not captured by current prenatal assessments. Our prior studies of T1D indicate that pancreas imaging may be an earlier predictor of diabetes than blood-based assays. 6 Pancreas imaging may similarly prove useful during pregnancy to identify mothers who would benefit from increased monitoring or interventions. High pancreatic fat in the early second trimester may be an early indicator of impaired glucose tolerance. By calculating a ratio between pancreas size and fat content, we developed a classifier that predicts GDM 10 weeks prior to diagnosis using standard-of-care glucose tolerance testing, albeit in a very small sample, thus replication is warranted. We found limited correlation between pancreas and liver fat content, suggesting that pancreas fat may reflect different aspects of the risk for adverse maternal outcomes than those associated with fatty liver. 22

The cost and availability of MRI will limit its use as a screening tool during pregnancy. However, maternal abdominal ultrasound is already performed during pregnancy and may be adapted to image pancreas size, particularly in those at risk of developing GDM, such as individuals with obesity or those with a family history of diabetes or GDM. Ultrasound has previously detected a smaller pancreas size in individuals with diabetes. 23 Thus, ultrasound may provide a convenient path for clinical adoption if pancreas imaging proves useful in predicting metabolic risk factors.

Strengths of this study include the use of MRI to assay multiple organ volumes and fat distribution concurrently, the frequent longitudinal follow-up with up to six MRI scans per participant, and the correlation with standard measurements of glucose metabolism. A primary limitation of this study is the limited number of participants who developed GDM, given that this study was designed among those with low GDM risk and thus underpowered to assess GDM risk. People with higher BMI values, and corresponding higher risk for GDM, were excluded due to the field of view size limitations of the MRI, so our results may not be generalizable to people with class II or class III obesity. Additionally, study participants were primarily White, which limits generalizability to other racial and ethnic groups that exhibited higher rates of GDM. The study of high-risk populations is needed to test whether pancreas size and fat fraction predict GDM in those at greatest risk. This cohort was enrolled during the COVID-19 pandemic, and there were missing MRI scans due to research shutdowns. Our first study visit occurred at approximately 15 weeks' gestation, and thus, we do not have pre-pregnancy pancreas measurements to establish dynamics in the first trimester. However, as beta cell mass expansion occurs primarily late in pregnancy, 24 it is likely that pancreas growth in the first trimester is limited. This ancillary study was initially designed to measure pancreas volume; consequently, the spleen and kidneys were not included in the field of view on all MRI scans. Nonetheless, we were able to measure these organ volumes on approximately 55% of MRI scans.

## Conclusion

In conclusion, our study demonstrates that pancreas size and composition change dynamically over the course of pregnancy and postpartum. The observed increase in pancreas volume during gestation, followed by a postpartum decline, suggests an adaptive response to the heightened insulin demand and commensurate beta cell proliferation/hypertrophy accompanying pregnancy. Associations between pancreas volume, fat content, and measures of glucose metabolism suggest that early alterations in pancreatic morphology may identify individuals at risk for impaired glucose tolerance. These findings support the potential value of longitudinal monitoring of pancreatic structure and composition during pregnancy and postpartum and may inform future research on strategies to prevent and manage GDM, especially in higher-risk populations.
